# Metric analysis of basal sphenoid angle in adult human skulls

**DOI:** 10.1590/S1679-45082014AO2933

**Published:** 2014

**Authors:** Dante Simionato Netto, Sergio Ricardo Rios Nascimento, Cristiane Regina Ruiz

**Affiliations:** 1 Centro Universitário São Camilo, São Paulo, SP, Brazil.

**Keywords:** Platybasia, Skull/anatomia & physiology, Measures, Adult, Human

## Abstract

**Objective:**

To analyze the variations in the angle basal sphenoid skulls of adult humans and their relationship to sex, age, ethnicity and cranial index.

**Methods:**

The angles were measured in 160 skulls belonging to the Museum of the Universidade Federal de São Paulo Department of Morphology. We use two flexible rules and a goniometer, having as reference points for the first rule the posterior end of the ethmoidal crest and dorsum of the sella turcica, and for the second rule the anterior margin of the foramen magnum and clivus, measuring the angle at the intersection of two.

**Results:**

The average angle was 115.41°, with no statistical correlation between the value of the angle and sex or age. A statistical correlation was noted between the value of the angle and ethnicity, and between the angle and the horizontal cranial index.

**Conclusions:**

The distribution of the angle basal sphenoid was the same in sex, and there was correlation between the angle and ethnicity, being the proportion of non-white individuals with an angle >125° significantly higher than that of whites with an angle >125°. There was correlation between the angle and the cranial index, because skulls with higher cranial index tend to have higher basiesfenoidal angle too.

## INTRODUCTION

At least three craniovertebral junction anomalies have been studied since the 18^th^ century: platybasia, basilar impression, and basilar invagination. There is great discrepancy in literature as to synonyms among the nomenclatures, which have distinct meanings and are commonly mistaken and used incorrectly.^(1-3)^ Platybasia results from increased obtuseness of the basal sphenoid angle of the cranium, with a tendency, therefore, to flattening of the anterior and posterior fossae of the cranium, resulting in a reduced posterior fossa.^(1,2,4,5)^ Basilar impression is the invagination of the bony contour of the foramen magnum inside the posterior fossa, in which the base of the skull assumes a cupulate form opposite to normal (convexobasia). The clivum is elevated and this anomaly is normally associated with the Arnold-Chiari syndrome.^(1,5)^ The term, “basilar invagination” is associated with an anomaly of primary development in which the spine is elevated and protracted relative to the base of the skull.^(1,6)^ Platybasia and basilar impression habitually appear together, but they can occur separately since isolated platybasia normally shows no symptoms.^(4)^ Some authors affirm that these anomalies have an ethnic character; others state that the influence is genetic.^(7-9)^ According to literature, the basal sphenoid angle varies greatly, with an interval from 103.5° to 152°, and a mean of 134°.^(8)^

## OBJECTIVE

Based on the statement that all these anomalies of the craniovertebral junction generate alterations of the base of the skull, and are, therefore, evaluated by the measurement of the amplitude of the basal sphenoid angle, the objective was to analyze the variations of these angles in supposedly normal adult human skulls and their relation with gender, age, ethnicity, and horizontal cranial index.

## METHODS

The angles were measured in 160 adult skulls from the Cranial Museum of the Department of Morphology, *Universidade Federal de São Paulo* which had come from indigents or unclaimed cadavers from the last 100 years; 89 skulls were from males (56%) and 85 from non-white individuals (53%).

The study only included skulls previously sectioned along the transverse plane and with complete exposure of the cranial cavity; non-sectioned skulls and those from individuals under 18 years of age were excluded. Measurements were made in July 2012.

**Figure 1:**
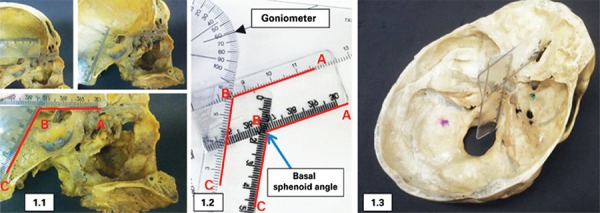
Method for measuring the basal sphenoid angle. (A) Medial sagittal plane of the cranium showing placement of the rulers in the anterior fossa (segment AB) and the clivus (segment BC), forming, with the intersection, the basal sphenoid angle ABC B; transport of the rulers fixed to the goniometer and measurement of angle C; representation of the true position of rulers on a cross-section

Two flexible millimeter rulers and a goniometer were used for the measurements. One of the rulers was placed parallel and in contact with the anterior fossa of the skull, using as reference points for its position the posterior extremity of the ethmoidal crest and the dorsum of the sella turcica. The second ruler was fixed on the anterior margin of the foramen magnum, in contact with and parallel to the clivus. The two rulers were then secured one to the other, and the angle formed by the intersection of the two was transferred to a goniometer. The angle was then measured (Figure 1).

For statistical analyses, we used Pearson’s χ^2^ tests, the Kruskal-Wallis test, Pearson’s coefficient of correlation, the Fi coefficient, Cramér’s coefficient, and the Marascuilo distribution procedure.

## RESULTS

The total mean of the angle measured was 115.41° (±8.45°). Among the female skulls, the mean was 115.56° (±8.83°), and among the males, 115.28° (±8.17°). Among the skulls from white individuals, the mean was 115.92° (±6.60°), and among non-whites it was 114.95° (±9.81°).

According to the data obtained in our survey, there was no statistical correlation between the value of the angle measured and gender, accepting the hypothesis that the value of the angles does not depend on the individual’s gender (Pearson’s χ^2^ value: 0.0838; α: 0.1; Kruskal-Wallis test: 3.5). Age of the skulls varied from 18 to 100 years (mean 43 years), and there was no statistically significant correlation between it and the value of the angle (Pearson’s correlation coefficient: 0.2).

However, ethnicity and the value of the angle measured were not independent variables (Pearson’s χ^2^: 5.7156; α: 0.1), although this association was statistically small (Fi coefficient: 0.189; Cramér coefficient: 0.189). Applying the Marascuilo distribution, it was noted that, statistically, the proportion of non-white individuals with an angle >25° was significantly greater than that of non-white individuals with an angle <125°, and that the proportion of white individuals with the angle between 115° and 125° was significantly greater than that of white individuals with an angle of <115° or >125°.

**Figure 2:**
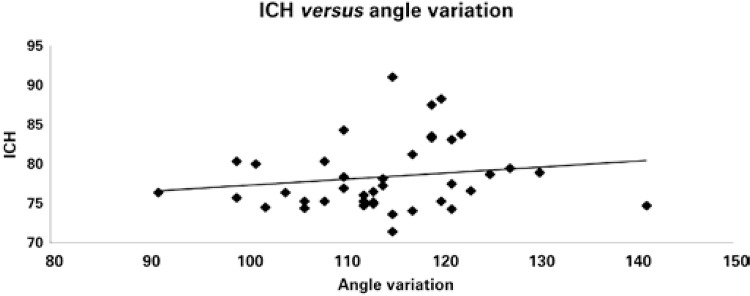
Tendency line between the increased horizontal cranial index and the increased value of the angle measured

Among the skulls studied, the horizontal cranial index was calculated in 40. Among the skulls classified as dolichocephalous, the mean angle measured was 112.16° (±10.58); among the mesocephalus, it was 114.46° (±9.43); and among the brachycephalus, it was 118.13° (±3.87). These two variables, horizontal cranial index and the angle measured, were not independent variables (Pearson’s χ^2^: 6.424; α: 0.05), although there was a weakly significant association between them (Fi coefficient: 0.401; Cramér coefficient: 0.283). Therefore, the skulls with a high horizontal cranial index showed a subtle tendency towards having a greater angle between the floor of the anterior fossa, the dorsum of the sella turcica, and the clivum (Figure 2).

## DISCUSSION

Just as in the study conducted by Royo-Salvador,^(4)^ in our sample there was no statistical correlation between the values of the basal sphenoidal angle and gender. We did, however, find a statistical association between the value of the angle measured and ethnicity, as did Caetano de Barros et al.^(5)^.

The values of the basal sphenoid angle found in literature vary a lot, as per McGreger:^(10)^ 103.5° to 131.5° for Boogard;^(11)^ 115° to 140° for List;^(12)^ 115° to 150° for Walsh et al.;^(13)^ 120° to 147° fora McGreger;^(10)^ 121.5° to 148.5° for Brailsford.^(14)^ Royo-Salvador^(4)^ found this angle in the interval from 115° to 140°. Our study obtained the interval from 89° to 141°.

As to platybasia, authors such as Royo-Salvador^(4)^ affirmed that there is platybasia when the basal sphenoid angle is more than 140°, but authors do not agree on the limit value for considering a skull platybasic. In our sample, only the cranium surpassed 140°, but due to the great discrepancy among values found in literature, this is not sufficient to affirm that this individual had a platybasic skull. Another important fact was that during these decades, experiments were carried out with many diverse materials and methods in which some projects were done with conventional X-rays and millimetered paper, others with mathematical formulas adapted for angle measurements, to more current methods such as magnetic resonance by means of sagittal slices.

The horizontal cranial index, defined as the centesimal relation verifiable between the transverse and maximum anteroposterior diameters of the skull when compared to the basal sphenoid angle, demonstrated in our sample a subtle tendency: when the index was greater, the basal sphenoidal angle also increased. It was noted in literature^(15)^ that the distribution of the skulls into types followed an ethnic parameter or one in reference to the individual’s biotype, which may be the answer as to why there is a correlation between the angle and ethnicity; yet, might it be suggested that there is a genetic influence for such differences? Authors such as Scoville and Sherman^(3)^ had already indicated that the familial component could be an important factor in these anomalies, and this was reaffirmed decades later by Pang and Thompson,^(7)^ who suggested that abnormalities in the Hox and Pax-1 genes could have an influence in many malformations of the craniovertebral junction. In our study, we do not have data for this comparison, since the project only focused on the anatomic field in question.

For an adequate evaluation of the craniovertebral relations, it is necessary to know in detail the anatomical structures that serve as parameter for the measurements of the craniometrical relations of the craniovertebral junction and of the adjacent anatomical structures, which in the live individual is very difficult, not only in terms of visualization, but also in terms of access. The diagnostic advances by image today allow not only the visualization of all the anatomical structures by magnetic resonance, but also the volumetric structure of the skull and its structures by means of 3-dimension computed tomography. Doubtless, these methods, associated with an excellent theoretical foundation, contribute towards the early diagnosis of platybasia as well as of other associated syndromes, since they are noninvasive methods, highly effective and accurate, that have extreme precision measuring tools.

In our sample, the mean of the angles was 115.41°, with no statistical correlation between the value of the angle and gender or age.

There was a statistical correlation between the value of the angle and ethnicity (χ^2^: 5.72) and between the angle and the horizontal cranial index (χ^2^: 6.42).

## CONCLUSION

Distribution of the basal sphenoid angle is the same between the genders, with a correlation between the angle and ethnicity in which the proportion of non-white individuals with an angle >125° was significantly higher than for white individuals with an angle >125°. There was a correlation between the angle and the horizontal cranial index, as skulls with a greater horizontal cranial index tended towards a greater basal sphenoid angle.
