# Supplementary material for: Metric analysis of basal sphenoid angle in adult human skulls
Source: Einstein (Sao Paulo). 2014 Jul-Sep;12(3):314–7. doi: 10.1590/S1679-45082014AO2933 (PMC4872942; doi:10.1590/S1679-45082014AO2933)
Supplement: Translation

# Translation: Análise métrica do ângulo basiesfenoidal em crânios de humanos adultos

**Authors:** Dante Simionato Netto, Sergio Ricardo Rios Nascimento, Cristiane Regina Ruiz

**Affiliations:** 1 Centro Universitário São Camilo, São Paulo, SP, Brasil.

**Keywords:** Platibasia, Crânio/anatomia & fisiologia, Medidas, Adulto, Humano

## Abstract

**Objetivo:**

Analisar as variações do ângulo basiesfenoidal em crânios de humanos adultos e sua relação com sexo, idade, etnia e índice crânico horizontal.

**Métodos:**

Os ângulos foram medidos em 160 crânios pertencentes ao Museu do Departamento de Morfologia da Universidade Federal de São Paulo. Utilizamos duas réguas milimetradas flexíveis e um goniômetro, tendo como pontos de referência para a primeira régua a extremidade posterior da crista etmoidal e o dorso da sela turca, e, para a segunda régua, a margem anterior do forame magno e o clivo, medindo o ângulo na intersecção das duas.

**Resultados:**

A média dos ângulos foi de 115,41°, não havendo correlação estatística entre o valor do ângulo e o sexo ou a idade. Houve correlação estatística entre o valor do ângulo e a etnia, e entre o ângulo e o índice crânico horizontal.

**Conclusão:**

A distribuição do ângulo basiesfenoidal foi a mesma entre os sexos, havendo correlação entre o ângulo e a etnia, e sendo a proporção de indivíduos não brancos com ângulo >125° significativamente maior que a de indivíduos brancos com ângulo >125°. Houve correlação entre o ângulo e o índice crânico horizontal, pois crânios com maior índice crânico horizontal tenderam a um ângulo basiesfenoidal maior.

## INTRODUÇÃO

Existem pelo menos três anomalias da junção craniovertebral que vêm sendo estudadas desde o século 18: a platibasia, a impressão basilar e a invaginação basilar. Há grande discrepância na literatura em relação à sinonímia entre as nomenclaturas, que possuem significados distintos e são comumente confundidos, sendo utilizados de maneira errônea.^(1-3)^ A platibasia resulta do aumento da obtusidade do ângulo basiesfenoidal do crânio, havendo, portanto, tendência ao nivelamento das fossas anterior e posterior do crânio, e resultando na diminuição da fossa posterior.^(1,2,4,5)^ A impressão basilar é a invaginação do contorno ósseo do forame magno no interior da fossa posterior, com a base do crânio ficando com forma cupular oposta à normal (convexobasia). O clivo é elevado e, normalmente, essa anomalia está associada à síndrome de Arnold-Chiari.^(1,5)^ O termo “invaginação basilar” é associado a uma anomalia do desenvolvimento primário, na qual a coluna vertebral encontra-se elevada e protraída em relação à base do crânio.^(1,6)^ A platibasia e a impressão basilar habitualmente se apresentam juntas, mas podem ocorrer separadamente, visto que a platibasia isolada normalmente não apresenta sintomas.^(4)^ Alguns autores afirmam que essas anomalias têm caráter étnico; outros afirmam que a influência é genética.^(7-9)^ De acordo com a literatura, o ângulo basiesfenoidal varia muito, sendo encontrado em um intervalo de 103,5° a 152°, com média de 134°.^(8)^

## OBJETIVO

A partir da afirmação de que todas essas anomalias da junção craniovertebral geram alterações na base do crânio e, por conseguinte, são avaliadas pela mensuração da amplitude do ângulo basiesfenoidal, o objetivo foi analisar as variações desse ângulo em crânios de humanos adultos supostamente normais e sua relação com sexo, idade, etnia e índice crânico horizontal.

## MÉTODOS

Os ângulos foram medidos em 160 crânios adultos, pertencentes ao Museu de Crânios do Departamento de Morfologia da Universidade Federal de São Paulo, oriundos de indigentes ou cadáveres não reclamados dos últimos 100 anos, sendo 89 crânios de homens (56%) e 85 de indivíduos não brancos (53%).

Foram incluídos apenas crânios previamente seccionados ao longo do plano transversal e com exposição completa da cavidade craniana; foram excluídos os crânios não seccionados e aqueles de indivíduos menores de 18 anos. As medições foram realizadas em julho de 2012.

Foram utilizadas duas réguas milimetradas flexíveis e um goniômetro para a mensuração dos ângulos. Uma das réguas era colocada paralela e em contato com a fossa anterior do crânio, tendo como pontos de referência para seu posicionamento a extremidade posterior da crista etmoidal e o dorso da sela turca. A segunda régua era fixada na margem anterior do forame magno, em contato e paralela ao clivo. As duas réguas eram, então, fixadas entre si, e o ângulo formado pela intersecção das duas era transportado a um goniômetro. O ângulo era, então, aferido (Figura 1).

**Figura 1:** Método de aferição do ângulo basiesfenoidal. (A) Figura em corte sagital mediano de crânio mostra a colocação das réguas na fossa anterior (segmento AB) e no clivo (segmento BC), formando, com sua intersecção, o ângulo basiesfenoidal ABC B; transporte das réguas fixadas ao goniômetro e mensuração do ângulo C; representação do posicionamento real das réguas no crânio em corte transversal

Para as análises estatísticas, utilizamos os testes de χ^2^ de Pearson, teste de Kruskal-Wallis, coeficiente de correlação de Pearson, coeficiente Fi, coeficiente de Cramér, e o procedimento de distribuição de Marascuilo.

## RESULTADOS

A média total do ângulo medido foi de 115,41° (±8,45°). Entre os crânios femininos, a média foi de 115,56° (±8,83°) e, entre os masculinos, de 115,28° (±8,17°). Entre os crânios de indivíduos brancos, a média foi de 115,92° (±6,60°) e, entre os não brancos, de 114,95° (±9,81°).

De acordo com os dados obtidos em nossa pesquisa, não houve correlação estatística entre o valor do ângulo medido e o sexo, aceitando-se a hipótese de que o valor dos ângulos independesse do sexo do indivíduo (valor χ^2^ de Pearson: 0,0838; α: 0,1; teste de Kruskal-Wallis: 3,5). A idade dos crânios estudados variou de 18 a 100 anos (média de 43 anos), e não houve correlação estatística significativa entre ela e o valor do ângulo (coeficiente de correlação de Pearson: 0,2).

Entretanto, a etnia e o valor do ângulo medido não foram variáveis independentes (χ^2^ de Pearson: 5,7156; α: 0,1), embora essa associação fosse estatisticamente pequena (coeficiente Fi: 0,189; coeficiente de Cramér: 0,189). Aplicando-se a distribuição de Marascuilo, observa-se que, estatisticamente, a proporção de indivíduos não brancos com o ângulo >25° foi significativamente maior do que a de indivíduos não brancos com o ângulo <125°, e que a proporção de indivíduos brancos com o ângulo entre 115° e 125° foi significativamente maior que a de indivíduos brancos com o ângulo <115° ou >125°.

Dentre os crânios estudados, 40 possuíam o índice crânico horizontal calculado. Entre os crânios classificados como dolicocéfalos, a média do ângulo medido foi de 112,16° (±10,58); entre os mesocéfalos, foi de 114,46° (±9,43); e entre os braquicéfalos, foi de 118,13° (±3,87). Essas duas variáveis, índice crânico horizontal e ângulo medido, não foram variáveis independentes (χ^2^ de Pearson: 6,424; α: 0,05), embora tenha havido associação pouco significativa entre elas (coeficiente Fi: 0,401; coeficiente de Cramér: 0,283). Assim, crânios com índice crânico horizontal alto tenderam sutilmente a um ângulo maior entre o assoalho da fossa anterior, o dorso da sela turca e o clivo (Figura 2).

**Figura 2:** Linha de tendência entre o aumento do índice crânico horizontal e o aumento do valor do ângulo medido

## DISCUSSÃO

Assim como no estudo conduzido por Royo-Salvador,^(4)^ em nossa amostra não houve correlação estatística entre os valores do ângulo basiesfenoidal e o sexo. Encontramos, porém, associação estatística entre o valor do ângulo medido e a etnia, do mesmo modo que Caetano de Barros et al.^(5)^.

Os valores do ângulo basiesfenoidal encontrados na literatura variam muito, de acordo com McGreger:^(10)^ 103,5° a 131,5° para Boogard;^(11)^ 115° a 140° para List;^(12)^ 115° a 150° para Walsh et al.;^(13)^ 120° a 147° para McGreger;^(10)^ 121,5° a 148,5° para Brailsford.^(14)^ Royo-Salvador^(4)^ encontrou esse ângulo no intervalo de 115° a 140°. Nosso estudo obteve intervalo de 89° a 141°.

Quanto à platibasia, autores como Royo-Salvador^(4)^ afirmaram que há platibasia quando o ângulo basiesfenoidal supera os 140°, porém não há concordância entre os autores sobre o valor limite para consideramos um crânio platibásico. Em nossa amostra, apenas um crânio ultrapassou os 140°, mas, devido à grande discrepância entre os valores encontrados na literatura, isso não é suficiente para afirmarmos que esse indivíduo possuía crânio platibásico. Outro dado importante foi que, durante essas décadas, os experimentos foram desenvolvidos com materiais e métodos bem diversificados, sendo alguns trabalhos realizados com radiografias convencionais e papel milimetrado, outros com fórmulas matemáticas adaptadas para a medição do ângulo, até métodos mais atuais, como a ressonância magnética, por meio de cortes sagitais.

O índice crânico horizontal definido como a relação centesimal verificável entre os diâmetros transverso e anteroposterior máximos do crânio, quando comparado ao ângulo basiesfenoidal, demonstrou, em nossa amostra, uma tendência sutil de que quando o índice fosse maior, o ângulo basiesfenoidal também aumentaria. Percebeu-se, na literatura,^(15)^ que a distribuição dos crânios em tipos segue um parâmetro étnico ou referente ao biotipo do indivíduo, o que pode responder o porquê da correlação entre o ângulo e a etnia; porém pode-se sugerir que há uma influência genética para tais diferenças? Autores como Scoville e Sherman^(3)^ já indicavam que o componente familiar poderia ser um fator importante nessas anomalias, e isso foi reafirmado décadas depois, por Pang e Thompson,^(7)^ que sugeriram que anormalidades nos genes Hox e Pax-1 podiam ter influência em muitas malformações na junção craniovertebral. Em nosso estudo, não temos dados para essa comparação, visto que o trabalho visou apenas ao campo anatômico da questão.

Para uma avaliação adequada das relações craniovertebrais, faz-se necessário um conhecimento detalhado das estruturas anatômicas que servem de parâmetro para as medidas das relações craniométricas da junção craniovertebral e das estruturas anatômicas adjacentes, o que, no crânio do indivíduo vivo, é difícil, em termos não só de visualização, mas também de acesso. Os avanços no diagnóstico por imagem permitem, hoje, não somente a visualização de todas as estruturas anatômicas, por meio da ressonância magnética, como também a estrutura volumétrica do crânio e suas estruturas por meio da tomografia computadorizada tridimensional. Sem dúvida alguma, esses métodos, associados a uma excelente fundamentação teórica, contribuem para o diagnóstico precoce da platibasia, bem como de outras síndromes associadas, pois são métodos não invasivos, de alta eficácia e acurácia, e que possuem ferramentas de medida de extrema precisão.

Em nossa amostra, a média dos ângulos foi de 115,41°, não havendo correlação estatística entre o valor do ângulo e o sexo ou a idade.

Houve correlação estatística entre o valor do ângulo e a etnia (χ^2^: 5,72) e entre o ângulo e o índice crânico horizontal (χ^2^: 6,42).

## CONCLUSÃO

A distribuição do ângulo basiesfenoidal é a mesma entre os sexos, havendo correlação entre o ângulo e a etnia, sendo a proporção de indivíduos não brancos com ângulo >125° significativamente maior que a de indivíduos brancos com ângulo >125°. Houve correlação entre o ângulo e o índice crânico horizontal, pois crânios com maior índice crânico horizontal tenderam a um ângulo basiesfenoidal maior.
